# 

**Published:** 1845-08

**Authors:** 


### CONTENTS

OF THE

### MEDICAL EXAMINER.

NEW SERIES—NO. VIII.—AUGUST, 1845.

### ORIGINAL COMMUNICATIONS.

History of Febrile Caloricity, xlvii. (Yellow Fever Dissection, cxliv.) By Bennet Dowler, M. D., of New Orleans, - - - - - 455

Notes of a Case of Ovarian Dropsy, with the appearances presented on Post Mortem Examination. By Edward R. Squibb, M. D., - 457

Case of Fungus of the Inferior Maxillary, successfully Treated. By D. Harrington, of Philadelphia, Dentist, - - - - - - 461

### CLINICAL LECTURES AND REPORTS.

### PHILADELPHIA HOSPITAL,

*Saturday, February* 8, 1845.

Clinic of Professor Dunglison.

Chronic Cutaneous Diseases, - - - - - - - - - 468

Stricture of some portion of the Intestinal Canal, - - - - 471

Epilepsy, - - - - - - - - - - - - - - - 472

### BIBLIOGRAPHICAL NOTICES.

Mental Maladies. A Treatise on Insanity. By E. Esquirol, Physician-in-Chief of the Maison Royale des Aliénés De Charenton, formerly Inspector General of the University, Member of the Royal Academy of Medicine, etc. Translated from the French, with additions, by E. K. Hunt, M. D., - - - - - - - - 476

On the Anatomy and Diseases of the Urinary and Sexual Organs; containing the Anatomy of the Bladder, and of the Urethra, and the treatment of the obstructions to which these passages are liable. By G. J. Guthrie, F. R. S., Surgeon to the Westminster Hospital, and to the Royal Westminster Ophthalmic Hospital, &c. &c. From the Third London Edition, - - - - - - - - 486

Lectures on the Theory and Practice of Surgery. By the late Abraham Colles, M. D., for thirty-four years Professor of Surgery in the Royal College of Surgeons in Ireland. Edited by Simon McCoy, Esq., F. R. C. S. I., - - - - - - - - - - - 498

Copland's Dictionary of Practical Medicine, Edited by Professor Lee, 499

The Medical Remembrancer, or Book of Emergencies; in which are concisely pointed out the immediate remedies to be adopted in the first moments of danger from poisoning, drowning, apoplexy, burns and other accidents. With the tests for the principal poisons, and other useful information. By Edward B. L. Shaw, M. R. C. S., & L. A. S., - - - - - - - - 499

### EDITORIAL.

The American Journal of Insanity, - - - - - - - - - 500

The New York Journal of Medicine and the Collateral Sciences, - 500

Medical College of the State of South Carolina, - - - - - 501

New Medical Journal in New Orleans, - - - - - - - - 501

Quackery, - - - - - - - - - - - - - - - - - 501

### RECORD OF MEDICAL SCIENCE.

A Case of Phrenitis resulting in Death, from the Inhalation of Sulphuric Ether. By Doct. J. Miller, of Louisville, Ky., - - - - 502
Hare-Lip in the Negro, - - - - - - - - - - - 505
Acclimating Fever of Liberia, - - - - - - - - - - 506
Medicine in Siam, - - - - - - - - - - - - - 506
On certain Differences in the Composition of the Blood in the Male and Female, - - - - - - - - - - - - - 507
Professor Berrutti on the Spontaneous Generation and Nature of the Spermatic Animalcules, - - - - - - - - - - 508
French and English opinions on the Treatment of Typhus, - - - - 509
The Actual Cautery successfully Employed in Gangrene of the Mouth, 510
Case of Dracuncules or Guinea-Worm, - - - - - - - 511
On the Periodic Discharge of Ova, and the Function of Menstruation, - 512
Case of Strangulated Congenital Hernia; occurring in a Child, six weeks old, and requiring Operation. By James Long, Esq., Surgeon to the South Dispensary, and Lecturer on Anatomy, Liverpool, - 513
Disease regarded as a False or Spurious Organisation. By Dr. Jahn, - 513
Preparation and Preservation of Ointments, - - - - - - 518

